# PASI: A novel pathway method to identify delicate group effects

**DOI:** 10.1371/journal.pone.0199991

**Published:** 2018-07-05

**Authors:** Maria K. Jaakkola, Aidan J. McGlinchey, Riku Klén, Laura L. Elo

**Affiliations:** 1 Turku Centre for Biotechnology, University of Turku and Åbo Akademi University, Turku, Finland; 2 Department of Mathematics and Statistics, University of Turku, Turku, Finland; Harbin Medical University, CHINA

## Abstract

Pathway analysis is a common approach in diverse biomedical studies, yet the currently-available pathway tools do not typically support the increasingly popular personalized analyses. Another weakness of the currently-available pathway methods is their inability to handle challenging data with only modest group-based effects compared to natural individual variation. In an effort to address these issues, this study presents a novel pathway method PASI (Pathway Analysis for Sample-level Information) and demonstrates its performance on complex diseases with different levels of group-based differences in gene expression. PASI is freely available as an R package.

## Introduction

The increasing relevance of personalized medicine makes it essential to have robust bioinformatic tools that enable sample-specific results. Pathway analysis is an example of a common approach in diverse biomedical studies [1, 2], for which the available tools do not typically support sample-level analysis, but provide only group-level pathway scores describing the difference in pathway activity between the test groups. However, multiple studies suggest that sample-level pathway scores can be applied to, for example, predicting disease phenotype or prognosis [3–7]. Based on the approach, pathway scores can represent for example pathway activity, enrichment of differentially expressed (DE) genes or deregulation compared to normal situation (i.e. activity in typical control sample). Due to the lack of pathway analysis methods providing sample-level results, studies have either calculated the sample-specific pathway scores manually without applying any existing pathway analysis tool, or done the predictions at gene-level using genes that appear in pathways highlighted by group-level pathway analysis [3–7]. This indicates a clear demand for automated pathway analysis methods that provide sample-specific pathway scores.

Another limitation with the current methods is their inability to handle data where the group effects on gene expression are moderate compared to natural individual variation. Yet the ability to analyze such challenging data is crucial for novel and subtle discoveries. Additionally, most of the available pathway methods require a list of DE genes as an input, making them by default unsuited to analyze data with only few, if any, such genes [8–10]. In our recent comparison study [11], some of the current pathway methods performed well in the study of clear cell renal cell carcinoma (where there was a large disease effect compared to individual variation), but with challenging Type 1 diabetes (T1D) data (which had a relatively small disease effect compared to individual variation) none of them performed sufficiently. The study also suggested that the methods utilizing pathway structures outperformed methods ignoring the structure.

Here we present a novel pathway method PASI (Pathway Analysis for Sample-level Information) as a way to address these outstanding issues. Therefore, PASI is designed 1) to provide a pathway deregulation score separately for each sample, 2) to utilize pathway structures, and 3) to highlight group-based differences in the expression even when the group effects are modest. To assess the performance of PASI with regard to these goals, we used six publicly available T1D datasets, including both recently diagnosed T1D cases and early state T1D cases before diagnosis, as well as three datasets on asthma, leukemia, and juvenile idiopathic arthritis (JIA) (see Table 1). Especially in T1D related datasets, most currently-available pathway tools have difficulties obtaining any findings, as the group effects are largely masked by individual variation before diagnosis.

**Table 1:** Summary of the nine analyzed datasets.

Dataset	Case+Control	Case group	Matched	Genetic risk	Insulin	Source	Accession
**T1D_1**	13+11	ndT1D	yes	yes	no	GEO	GSE30211
**Sero_1**	19+17	sero	yes	yes	no	GEO	GSE30211
**T1D_2**	43+18	ndT1D	no	no	yes	GEO	GSE9006
**T1D_3**	12+10	ndT1D	no	no	yes	GEO	GSE55098
**T1D_4**	49+88	ndT1D	no	yes	yes	AE	MTAB1724
**Sero_2**	17+17	sero	yes	yes	no	AE	MTAB1724
**Asthma**	17+18	asthma	no	-	-	GEO	GSE27011
**JIA**	85+16	JIA	no	-	-	GEO	GSE79970
**Leukemia**	41+11	leukemia	age	-	-	GEO	GSE22529

Column “Case+Control” describes the sample size and column “Case group” the case group used in the dataset; ndT1D refers to newly diagnosed Type 1 diabetes (T1D) and sero to the first measurement after seroconversion but before diagnosis. Columns “Matched”, “Genetic risk” and “Insulin” indicate whether control samples were matched to case samples by age and gender, whether all individuals in the study had a genetic risk of T1D, and whether the case samples had already been treated with insulin, respectively. The two last columns, “Source” and “Accession”, provide the origin of the data (GEO refers to Gene Expression Omnibus [14] and AE to ArrayExpress [15]) and its accession id.

We demonstrate the ability of PASI to highlight the differences between the sample groups as compared to two other state-of-the-art pathway methods providing results in similar format, Pathifier [12] and PerPAS [13]. To our knowledge, Pathifier and PerPAS, are the only available pathway software that provide pathway deregulation scores for all samples separately. To assess the utility of pathway information on the results, we also compared PASI to a corresponding gene-level approach. Finally, we investigated the impact of sample size on the results and demonstrated the robustness of PASI against uncertainty in pathway structures. The PASI R package is freely available at https://www.btk.fi/research/computational-biomedicine/.

## Materials and methods

### Datasets

PASI was tested using nine different datasets, which contain data on T1D, asthma, Juvenile Idiopathic Arthritis (JIA), or leukemia. The nine datasets were based on seven studies: GSE30211, GSE9006, MTAB1724, GSE55098, GSE27011, GSE79970, and GSE22529, which are publicly available in the Gene Expression Omnibus (GEO) [14] or in ArrayExpress [15]. Normalized data were downloaded from the databases. Any data obtained that was already log-scaled was converted back to non-log scale for our analyses. The datasets are summarized in Table 1 and full lists of used samples are available in S1 Table to assure reproducibility.

In T1D related datasets, the case samples were either recently diagnosed T1D patients or individuals with seroconversion i.e. auto-antibody detection but before diagnosis. Datasets including case samples before diagnosis were expected to be more challenging to analyze than those after diagnosis, with smaller disease-based changes in gene expression. JIA data represented RNA-seq data in contrast to the other microarray datasets and leukemia data represented data with large disease effect. PASI is designed to detect moderate differences between sample groups so cancer is not in its main focus. However, as both Pathifier and PerPAS have been validated with cancer data, we tested also PASI on leukemia.

Dataset GSE30211 [16] included time series data from children with T1D-associated HLA genotype i.e. genetic risk of T1D. The few samples associated with enterovirus infection [17] were excluded for this study. We utilized the data in two separate comparisons. In the first comparison, the case group consisted of 13 samples with recently diagnosed T1D and the control group of 11 healthy control samples. In the second comparison, the case group included 19 samples right after seroconversion and 17 healthy control samples. These two comparisons are further referred to as T1D_1 and Sero_1, respectively.

In the dataset GSE9006 [18] we compared 43 newly diagnosed T1D patients to 18 healthy controls. Control samples labeled with HIDDM or INF were excluded from the study and only chip A measurements were used. This comparison is further referred to as T1D_2.

In the dataset GSE55098 [19] we used the 12 T1D samples as cases and the 10 non-related healthy samples as controls in our comparison T1D_3.

Dataset MTAB1724 [20] included both time series and single time point data. Again, we used the dataset in two comparisons. In the first comparison, we compared 49 newly-diagnosed T1D samples to 88 healthy control samples. In the second comparison, we compared 17 seroconverted samples to 17 healthy control samples. These two comparisons are referred to as T1D_4 and Sero_2, respectively.

From the dataset GSE27011 [21] we compared 17 patients with serious drug resistant asthma to 18 healthy controls. This comparison is referred to as Asthma.

From the dataset GSE79970 [22] we utilized all 85 case samples with Juvenile Idiopathic Arthritis and 16 healthy control samples in our comparison named as JIA.

In the dataset GSE22529 [23] we compared measurements from 41 Chronic Lymphocytic Leukemia tumor samples and 11 age matched controls. Only chip U133A measurements were used. This dataset is referred to as Leukemia.

### Pathway analysis for sample-level information PASI

**Fig 1:**
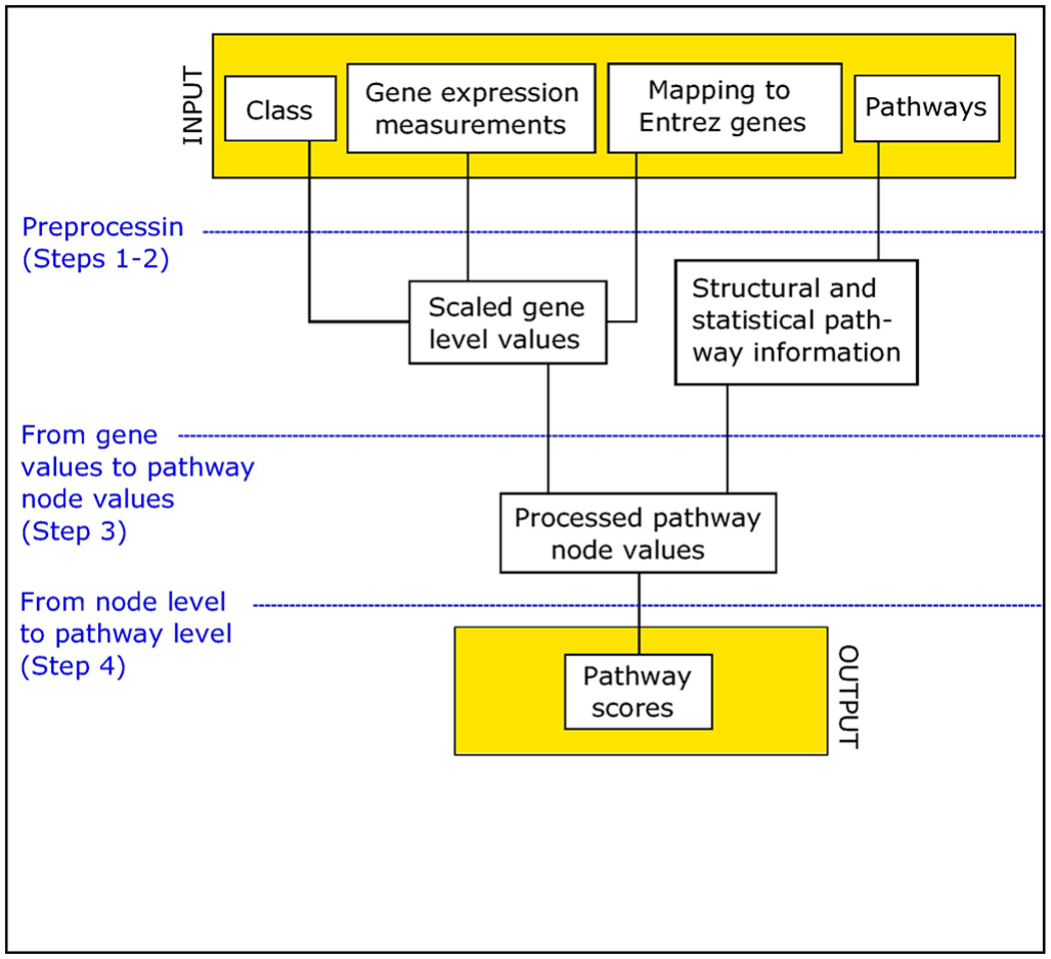
Workflow of the pathway analysis for sample-level information PASI. The method involves four steps described briefly above and in more detail in S1 Appendix.

In this section, we present an overview of the working principles of PASI. More details about file formats are available in the user manual provided with the R package and detailed description of the method is provided in S1 Appendix. Fig 1 provides a schematic illustration of the PASI workflow.

Version 1.0.0 of PASI requires as input

1. Gene expression data matrix including preferably at least 15 healthy control samples
2. Mapping from gene/probe IDs to Entrez IDs
3. Vector of labels indicating case and control samples and
4. KEGG pathways [24]

In the terminology used here, a node is a functional unit of a pathway, typically a gene product, and a relation is a directional interaction between two nodes. The final pathway scores of PASI describe similarity of pathway’s activity to normal activity (low score: normal, high score: abnormal) rather than actual pathway activity. The steps of the PASI workflow are:

**Step 1** Preprocess the gene expression data to produce scaled, noise-filtered gene-level values.
**Step 2** Preprocess the pathway files from KEGG pathway database to extract information about their structure and composition.
**Step 3** Transform the gene expression values from Step 1 and pathway information from Step 2 to pathway node values describing their similarity to the activity in normal situation.
**Step 4** Calculate the sample-specific pathway deregulation scores using pathway node values from Step 3 and importance of the nodes defined using pathway information from Step 2.

### Test design

To demonstrate the utility of PASI, we tested it from different perspectives as detailed below.

#### Comparison to other approaches

To evaluate the performance of PASI in comparison to the other pathway approaches, Pathifier, and PerPAS, we used logarithmic ratios of average FDR values between real and mock data (later denoted as FDR ratio). First, we compared pathway scores of case and control samples in each pathway using Wilcoxon test. The resulting p-values were then converted to FDR values and their average ($\bar{\text{FDR}}$) over pathways was used to calculate FDR ratio. The $\bar{\text{FDR}}$ from the real data describes how well pathway scores distinguish between the sample groups. In the artificial mock data, no differences between the sample groups are expected, and therefore the FDR values should be high. Artificial mock datasets were generated from real dataset by randomly dividing the real case and control samples into two equally sized artificial groups. Ten randomized mock datasets were generated from each dataset. The FDR ratio was formally defined as:

$$\begin{array}{c} FDRratio\left(data\right)=-log\left(\frac{\bar{\text{FDR}}\left(data\right)}{\frac{1}{10}\sum_{i=1}^{10}\bar{\text{FDR}}\left(mock\_data_{i}\right)}\right). \end{array}$$

Here *data* is the real dataset including samples from two groups (case and control) and *mock*_*data*_*i*_ refers to *i*^*th*^ randomized mock data. The higher the FDR ratio, the better a method distinguishes real differences from random noise.

Besides comparing PASI to Pathifier and PerPAS, we tested the impact of pathway information in PASI algorithm as compared to simply scaling the gene-level expression values similarly as in PASI. This was done by comparing the FDR ratio of PASI to FDR ratio of the gene-level approach.

#### Effect of sample size

To test the effect of sample size on the results, we reduced either the number of control samples or the number of case samples in the largest dataset T1D_4. Ten datasets were randomly generated at each tested sample size between 5 and 30. Similarly, we generated mock datasets by dividing the 88 control samples from the data into two artificial groups without any real differences. Again, ten datasets were randomly generated at each sample size between 5 and 30.

#### Uncertainty in pathway structure

To test the effect of uncertainty in pathway structure, we added different amounts of “uncertainty” (missing nodes or relations) to the well-known MAPK signaling pathway in the dataset T1D_1, where it originally was among top detections with PASI, based on the difference in the median pathway scores between the case and control samples. Uncertainty was simulated by randomly removing 5, 10, 20, 30, 40 or 50 percent of relations or nodes from the pathway, 100 times for each proportion. More specifically, we investigated whether the pathway remained in the top 25 detections (approximately 10% of tested pathways) despite uncertainty in the structure (simulated by removing nodes/relations from the pathway).

#### Biological relevance of the findings

Two pathways previously suggested to be enriched with biologically-relevant genes in T1D [16, 20, 25], Interferon signaling (referred to as IFN pathway) and Activation of IRF by cytosolic pattern recognition receptors (referred to as IRF pathway), were available in IPA (QIAGEN’s Ingenuity Pathway Analysis: www.qiagen.com/ingenuity, accessed 13-September-2016). For testing, we manually constructed IFN and IRF pathways into the KEGG pathway format [24], gave them artificial KEGG-alike IDs (hsa06000 for IFN pathway and hsa07000 for IRF pathway), and analyzed them together with KEGG pathways using PASI. We then investigated their detection ranks in the six T1D datasets, defined similarly as above.

## Results

We tested the performance of PASI in nine datasets, each including a control group of healthy individuals and a case group of either recently diagnosed T1D patients (datasets marked with the prefix T1D), measurements after seroconversion, but before T1D diagnosis (datasets marked with the prefix Sero), severe asthma (dataset Asthma), JIA or leukemia. Since it would be misleading to use a single metric to estimate the usefulness of a method, we assessed the performance of PASI in a variety of ways including

1. comparison of PASI to the closest state-of-the-art pathway methods Pathifier and PerPAS,
2. investigation of the effects of gene-level scaling and adding pathway information in PASI algorithm,
3. assessment of the effect of sample size on the pathway results,
4. assessment of the effect of uncertainty in pathway structures on the results, and
5. biological relevance of the results.

### Performance of PASI, Pathifier and PerPAS

We first compared PASI results to Pathifier [12] and PerPAS [13] results. To measure the ability of PASI, Pathifier and PerPAS to distinguish differences between sample groups from random variation, we used FDR ratio between real and mock data as an evaluation metric (see Section Test design for details). FDR ratios together with $\bar{\text{FDR}}$ values from real (FDR) and average mock (M_FDR) data are listed in Table 2.

**Table 2:** Comparison of PASI, Pathifier and PerPAS.

	FDR ratio				FDR				M_FDR			
Dataset	PASI	Pathifier	PerPAS	Gene	PASI	Pathifier	PerPAS	Gene	PASI	Pathifier	PerPAS	Gene
**T1D_1**	1.26	0.08	1.14	-0.10	0.33	0.49	0.35	1.00	0.80	0.51	0.77	0.93
**T1D_2**	2.45	2.18	1.73	0.17	0.13	0.09	0.22	0.84	0.71	0.41	0.74	0.95
**T1D_3**	3.40	1.44	1.53	0.07	0.06	0.15	0.20	0.88	0.59	0.41	0.57	0.93
**T1D_4**	8.60	9.47	4.80	2.58	0.002	0.001	0.03	0.16	0.95	0.83	0.95	0.97
**Sero_1**	1.62	0.08	0.89	-0.06	0.23	0.50	0.38	0.96	0.69	0.53	0.70	0.92
**Sero_2**	0.89	0.43	0.49	-0.11	0.32	0.17	0.44	0.98	0.60	0.23	0.62	0.91
**Asthma**	1.75	1.38	1.02	0.27	0.23	0.17	0.37	0.80	0.79	0.45	0.75	0.96
**JIA**	4.07	3.10	3.76	1.20	0.04	0.08	0.06	0.36	0.69	0.72	0.81	0.83
**Leukemia**	4.04	3.90	2.56	1.03	0.05	0.03	0.13	0.45	0.75	0.38	0.75	0.93

FDR ratio and average $\bar{\text{FDR}}$ of real (FDR) or mock data (M_FDR) in the nine datasets tested using PASI, Pathifier, PerPAS and the gene-level approach. The results with the highest FDR ratio in each dataset are underlined.

For most of the datasets processed, PASI had consistently higher FDR ratios than Pathifier and PerPAS (Table 2). All methods had higher FDR ratios in datasets with diagnosed T1D case samples (1.26-8.60 for PASI, 0.08-9.47 for Pathifier, and 1.14-4.80 for PerPAS) than in the more challenging datasets with seroconversion case samples before T1D diagnosis (0.89-1.62 for PASI, 0.08-0.43 for Pathifier, and 0.48-0.89 for PerPAS). As compared to PASI, Pathifier had typically quite similar $\bar{\text{FDR}}$ values in the real comparisons, but smaller average $\bar{\text{FDR}}$ values in the mock comparison indicating overfitting. PerPAS had higher $\bar{\text{FDR}}$ values in the real data.

### Pathway-level versus gene-level analysis

The FDR ratios in different datasets were also calculated directly based on the scaled gene expression profiles (scaling explained in S1 Appendix Section 1) to see if adding pathway information to the workflow improved distinguishing the sample groups.

**Fig 2:**
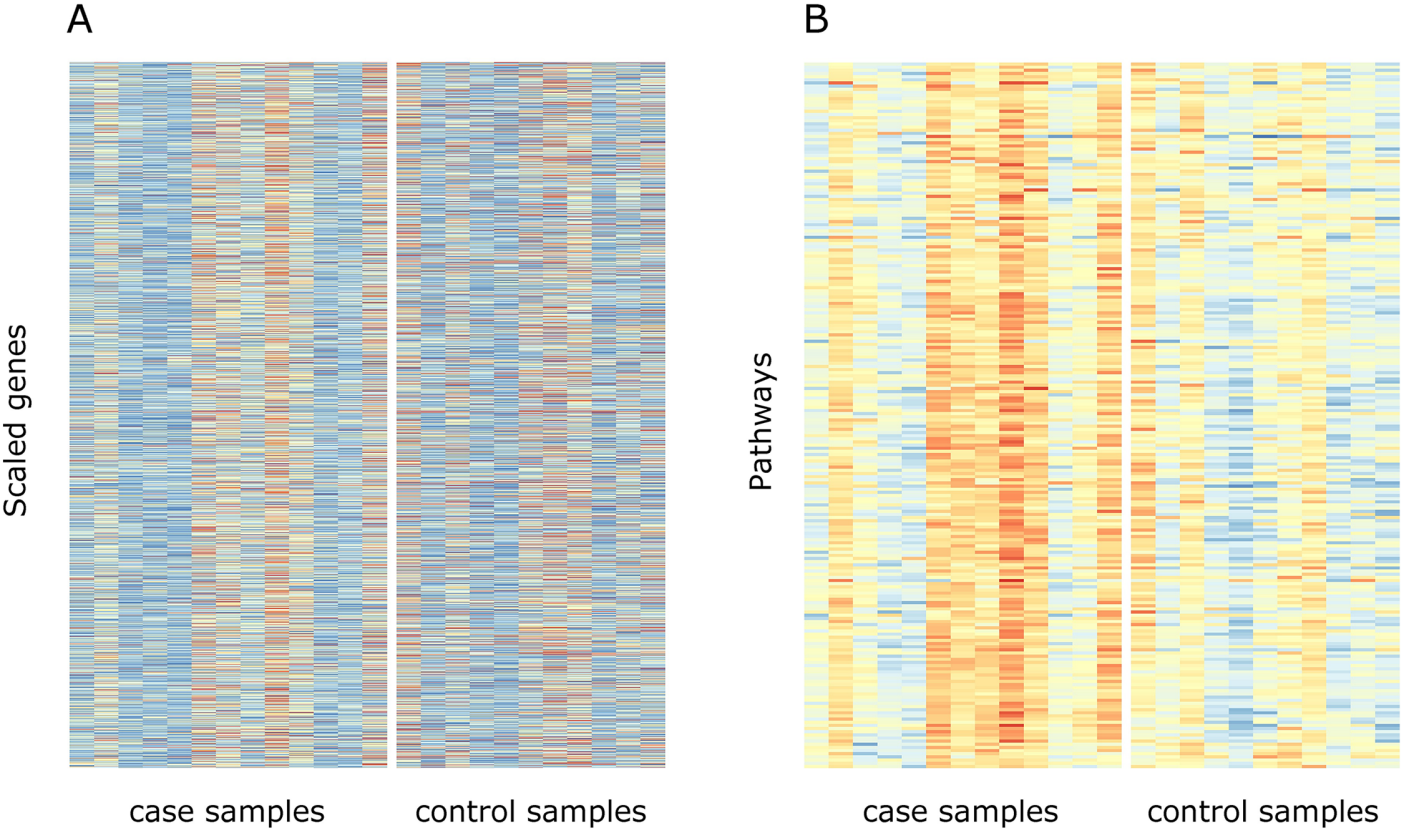
Gene values and pathway scores. Representative example of (A) gene- and (B) pathway-level results on dataset T1D_1. Columns are samples and rows are (A) genes or (B) pathways. The gene expression values for the gene-level analysis were scaled as in PASI. The pathway scores were calculated with PASI. Blue colour indicates a low value and red colour a high value.

In all of the tested datasets, the pathway-level results were demonstrably better than the gene-level results (Table 2). The low FDR ratios of the gene-level approach occurred due to high $\bar{\text{FDR}}$ values in real data (0.16-1.00) rather than low average $\bar{\text{FDR}}$ in mock data (0.91-0.97) indicating that the scaling of PASI alone does not distinguish the sample groups, but pathway information is needed. Fig 2 illustrates the difference between pathway-level analysis and gene-level analysis in the dataset T1D_1, showing how the group effect is more visible at the pathway-level compared to the gene-level.

### Effect of sample size

We next tested the effect of sample size on the accuracy of the results by reducing the number of samples in the largest dataset T1D_4 (49 cases, 88 controls). Different sample sizes between 5 and 30 were tested with ten randomly-generated data at each size.

**Fig 3:**
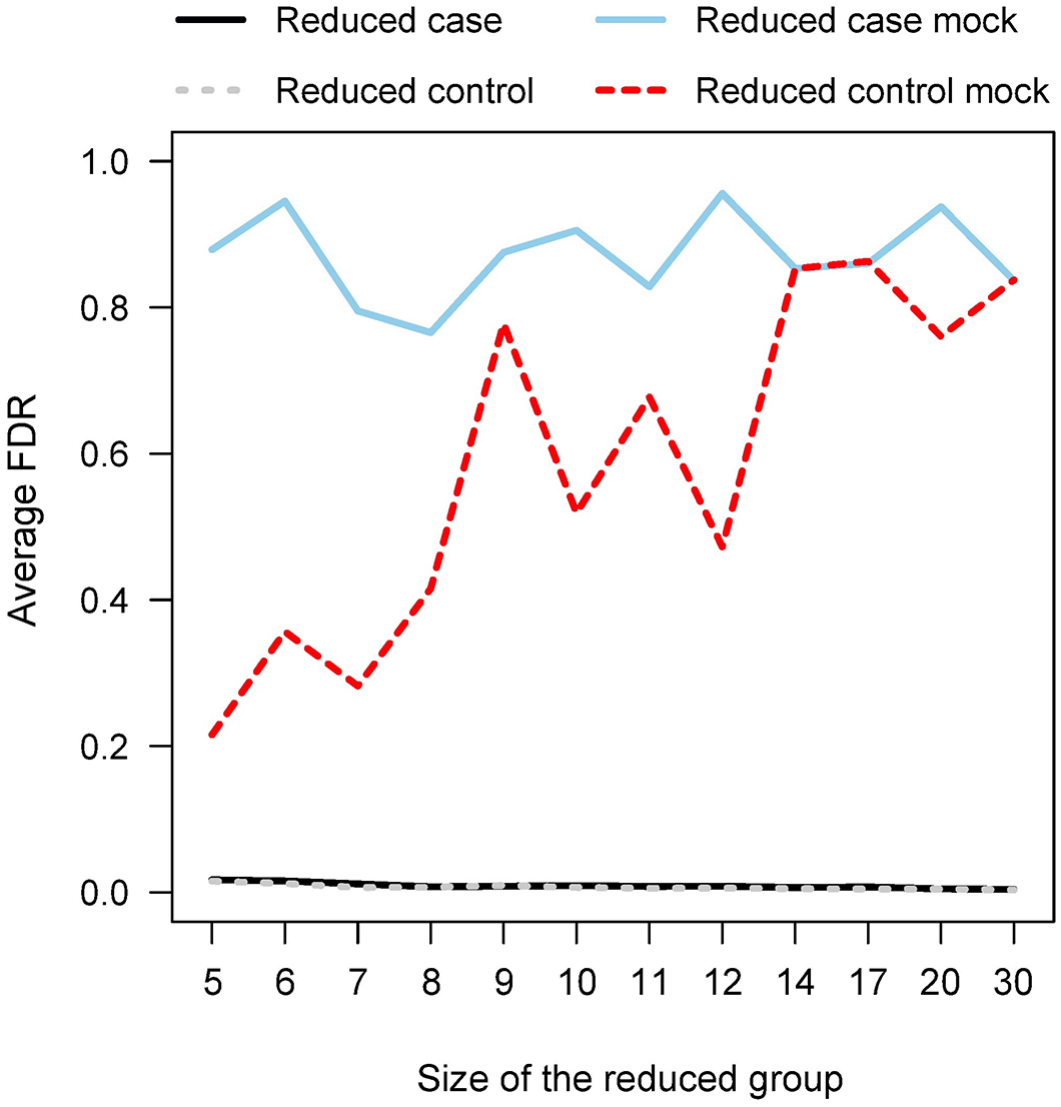
Sample size effect. Effect of sample size (x-axis) on the average $\bar{\text{FDR}}$ (y-axis) in data T1D_4. The black solid line illustrates average $\bar{\text{FDR}}$ when all control samples were used, but case samples were limited to a randomly selected subset (x-axis). Similarly, the gray dotted line illustrates average $\bar{\text{FDR}}$ when all case samples were used, but number of control samples was reduced. The blue solid line and the red dashed line describe the average $\bar{\text{FDR}}$ in mock data when the number of case or control samples were reduced, respectively. High $\bar{\text{FDR}}$ is expected from mock data as there is no difference between the test groups. Ten random subsets of samples for each sample size were used to obtain the average $\bar{\text{FDR}}$ values.

Overall, the reduction of the sample size did not have a large negative impact on the findings (Fig 3, black solid line and gray dotted line). Instead, the average $\bar{\text{FDR}}$ remained low (lower than 0.02) with all tested sample sizes regardless of whether the number of case samples or the number of control samples was reduced. However, with a small number of control samples the $\bar{\text{FDR}}$ values were low also in randomized mock data (Fig 3, red dashed line) indicating overfitting. When reducing the number of case samples in the mock data, such overfitting was not observed (Fig 3, blue solid line). Therefore, we recommend the use of at least 15 control samples.

### Effect of uncertainty in pathway structure

One critisism of pathway analyses is that often the pathway structure is not completely known [26, 27]. To simulate this, we reduced the large and well-known Mitogen associated protein kinase (MAPK) signaling pathway by randomly excluding different proportions of its nodes or relations (5-50%) and investigated whether the removal affected the detection of the pathway among the top 25 pathways in the dataset T1D_1. When the full, known pathway structure was used, MAPK signaling was among the top pathways in the dataset T1D_1. The random selection of relations or nodes to be removed from the pathway was repeated 100 times for each proportion. Removing nodes was expected to have a greater impact on the results than removing relations, because removal of a node requires removal of all relations involving the node as well.

**Fig 4:**
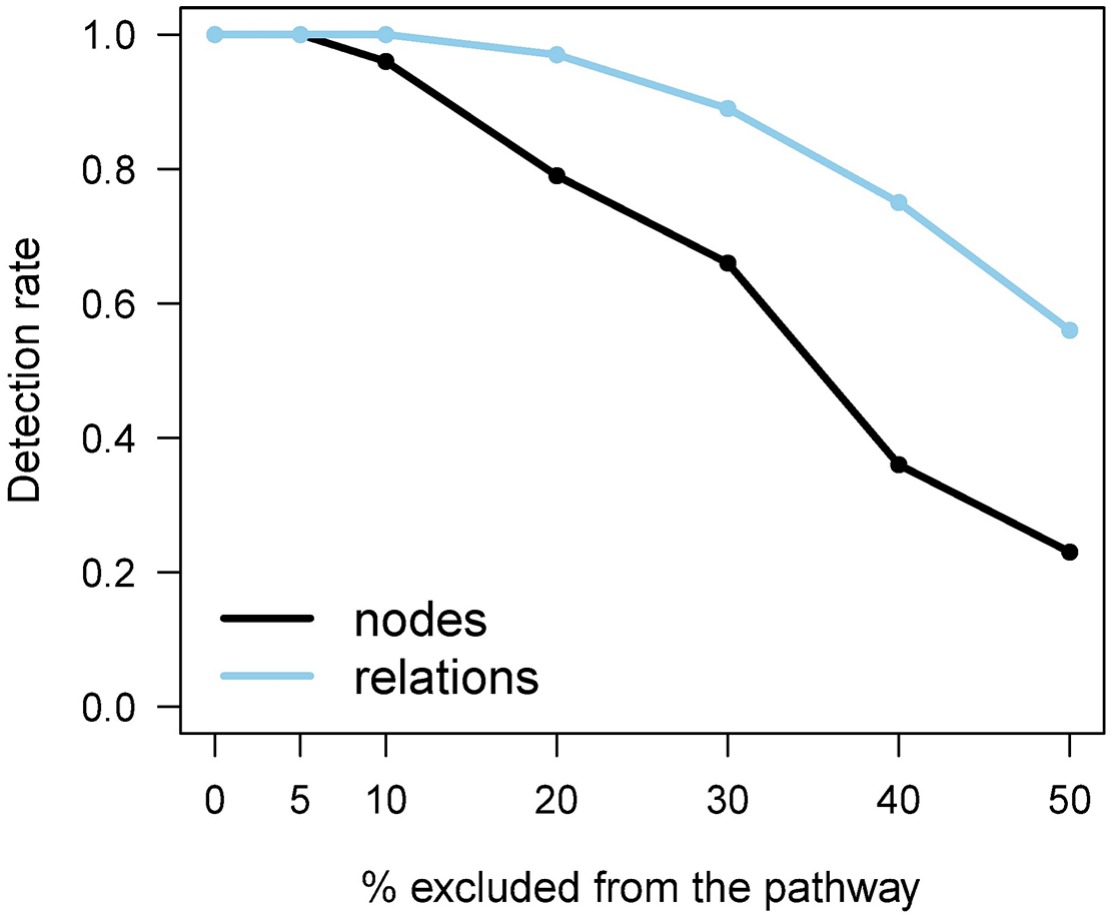
Effect of structural uncertainty. Rate of detection (y-axis) of the MAPK signaling pathway among the top 25 pathways when removing different percentages (x-axis) of nodes or relations from it. Reduction was done separately for nodes (black line) and relations (blue line). 100 random reductions for each proportion were used to calculate the detection rates in dataset T1D_1.

Encouragingly, small reductions in the MAPK signaling pathway had no effect on detecting it among the top pathways in the T1D_1 data; it remained consistently within the top 25 detections when the proportion of removed nodes or edges was moderate (Fig 4). As expected, when we increased the proportion to be excluded, the effect on detection rate also increased and excluding nodes had a higher impact on the detection rates than excluding relations.

### Biological interpretation in T1D datasets

Whilst PASI was designed to detect overall differences between sample groups, it was also desired that it should return pathway results that are of relevance to the disease in question. Since the tested T1D datasets included case samples from different disease states, age groups and insulin treatments (Table 1), it is unlikely that one pathway would consistently be reported for all these subgroups. Therefore, we investigated median ranks of the pathways across the six tested T1D datasets (S2 Table). It was reassuring to see that well-known T1D-related pathways were found within the top 30 pathway detections by PASI (corresponding to the top 14% of pathways). The top 30 was chosen for this section as the pathway “hsa04910—Insulin signaling pathway” was ranked 28, and this pathway is clearly implicated in T1D.

As with any pathway analysis, it is to be expected that pathways in this top list may be present due to (a) direct relevance to the biological phenomenon (in this case T1D), (b) overlap with (a), or (c) no (current) known relevance to the phenomenon in question (S2 Table). In order of median ranks, the top detection here was “hsa04917—prolactin signaling”. Prolactin (PRL) is known to directly increase *β*-islet cell insulin secretion [28] and PRL level has been recently linked to deficient glucose regulation and the state of diabetes itself [29]. Two very general pathways, the Mitogen associated protein kinase (MAPK) signaling pathway (hsa04010) and Rat sarcoma (Ras) pathway (hsa04014), were among the top 30 detections. These pathways are associated with numerous processes in human body, some of them related to diabetes [30–36]. Besides these general pathways, multiple immune related pathways were also detected, such as the interferon (IFN) and interferon regulatory factor (IRF) pathways (as detailed in Section Test design). This is in line with the observation that children at risk of T1D have been shown to have a distinct interferon signature before seroconversion [16, 20]. Concerning the IFN pathway, in the different T1D datasets, different genes contributed to the enrichment for the pathway, underscoring the advantage of pathway analysis compared to gene-level analysis. These different genes and their contributions are shown in S1 Fig. In addition to interferon pathways, several other canonical immune related pathways were among these top detections, such as Toll-like (hsa04620), B-cell (hsa04662) and FceR receptor pathways (hsa04664).

## Discussion and conclusions

We introduced here a new pathway analysis tool, PASI, and illustrated its competitive performance. The results were good (FDR ratio ≥ 1.26) in all tested datasets with diagnosed T1D, asthma, JIA or leukemia case samples and relatively good (FDR ratio ≥ 0.89) even in the challenging data with case samples before T1D diagnosis. Although, with large datasets including over 60 samples (T1D_4 and T1D_2) Pathifier performed very well, sample size had a notable impact on the Pathifier results and it seemed to require more samples to perform reliably compared to PASI. While PerPAS did not have the top performance in any of the tested datasets, it outperformed Pathifier in most of the small (under 60 samples) datasets. With PASI, we recommend using at least 15 control samples to keep the false positive effect moderate.

Importantly, PASI was not sensitive to small uncertainty in pathway structure (e.g. where missing node / relation percentage did not exceed 10%), which is critical as the pathway structures represent only the current knowledge instead of the absolute truth, and are frequently updated as more information becomes available. As pathway topologies are updated with experimental evidence, it is expected that methods such as PASI that use pathway topology only increase in their utility. Our previous observations about group-level pathway methods suggest that currently, methods applying structural information in relatively general level outperform methods that apply it in detail or do not apply it at all [11]. Therefore, we implemented PASI to utilize pathway structures, but only in rough level via feedback (see S1 Appendix).

Overall, our results demonstrated the utility of pathway-level analysis in identifying moderate group effects. Results obtained without using pathway information did not distinguish the sample groups at all (FDR ratio below or close to 0) in most of the tested datasets (Table 2). Adding the pathway information to the analysis clearly improved the performance (FDR ratio ≥ 0.89). Visualization of the IFN pathway (S1 Fig) illustrated the advantages of pathway-level analysis over gene-level analysis by showing how different genes alter the pathway in different studies. This indicates difficulties in finding consistently differentially expressed genes. Although the focus of PASI is in capturing group-based differences between samples, biologically-relevant pathways were consistently among the top detections.

Some of the original papers introducing the data utilized in this study included pathway analysis. Our PASI analysis reproduced many, but not all, of the original findings and introduced several new pathway detections as well. Some examples of the reproduced findings were N-Glycan biosynthesis from dataset Asthma, RIG-I-like receptor signaling pathway from datasets T1D_1 and Sero_1, apoptosis pathway from dataset T1D_2 and Wnt signaling pathway from Leukemia. As reported in section Biological interpretation in T1D datasets, pathway Prolactin signaling was an example of a PASI finding detected systematically from different T1D related datasets, but not by the original publications.

In PASI, we chose to use pathway deregulation scores describing similarity to normal rather than scores describing pathway activity. Whilst the pathway activity scores can provide more information than deregulation scores, their weakness is unsuitability for pathways with inhibiting relations. Inhibiting relations in a pathway indicate that not all nodes are highly expressed when the pathway is active. Due to structures like inhibitor of an inhibitor node, it is highly nontrivial task to computationally define which pathway nodes should be highly expressed when the pathway is active. While on average the portion of inhibiting relations in KEGG pathways is currently about 12%, in several pathways more than half of the relations are inhibiting, making those pathways unsuited for simple pathway activity scoring methods assuming that high expression of the nodes indicates high pathway activity.

Besides developing better pathway tools, pathway-based studies can be improved by upgrading pathway databases. A number of databases exist with their own focus and special characteristics that make them suited for studies of different topics. However, at the moment, very few pathway databases are free, provide directed interactions, and offer the pathways in a computer-friendly format. The possibility to utilize a larger range of databases may have the potential to significantly enhance pathway analysis and all its applications.

PASI enables the identification of group effects even if this effect is small in comparison to individual variation. The intended main applications of PASI lie in studies related to personalized medicine and predictive modeling. PASI is less suited for mechanistic studies where the focus is on detecting few pathways which differ in their activity between the test groups or revealing information about mechanisms behind the conditions. Since PASI is designed to be robust to limited uncertainty in pathway structures, it is currently not sensitive to detect whether assumed structures are incomplete, which is a different research topic with its own methods [37].

## Supporting information

S1 Table Lists of used sample ids. (XLSX)

S2 Table Ranking of pathways. (XLSX)

S1 Appendix Supplementary text. Detailed description of PASI workflow.
(PDF)

S1 Fig IFN pathway visualization. (PDF)
