# Supplementary material for: PASI: A novel pathway method to identify delicate group effects
Source: PLoS One. 2018 Jul 5;13(7):e0199991. doi: 10.1371/journal.pone.0199991 (PMC6033442; doi:10.1371/journal.pone.0199991)
Supplement: S1 Appendix — Detailed description of PASI workflow. (PDF) [file pone.0199991.s003.pdf]

Supplementary Text

PASI: A Novel Pathway Method to Identify Delicate Group Effects

Maria K. Jaakkola*[1,2], Aidan J. McGlinchey[1], Riku Klén[1] and Laura L. Elo[1]

[1]Turku Centre for Biotechnology, University of Turku and Åbo Akademi University
[2]Department of Mathematics and Statistics, University of Turku

***Correspondence:** Maria K. Jaakkola
Email: makija@utu.fi
Phone: +358 2 9450 3796
Adress: Turku Centre for Biotechnology, Tykistökatu 6, FI-20520 Turku, Finland

1 Detailed description of PASI workflow

1.1 Preprocessing gene expression data (Step 1)

Step 1 preprocesses the gene expression data. First, measurements are converted to genes (Entrez id). In the context of microarrays, probes mapping to multiple genes are excluded and in case multiple probes map to one gene, the probe with the highest overall intensity is chosen to represent the gene.

After converting the input measurements into gene-level values, the noise is filtered out. Using our custom made algorithm, noise level is first detected separately for each sample by searching for a point in density plot where two normal distributions representing noise and real signal merge. The merging point is a local minimum in the density plot. The median noise level over the samples is used as a cutoff for lowly expressed genes. Each gene with an expression level lower than the cutoff in at least half of the samples in both test groups is filtered out as unexpressed. Also genes with less than four measured control samples are excluded from the data.

After filtering, the remaining data is scaled. The scaled expression value of a gene g in sample i, x_{gi} is calculated as

$$z_{gi} = \frac{x_{gi} - \text{mean}(x_{gi}|i \in C^*(g))}{\text{sd}(x_{gi}|i \in C^*(g))},$$

where $C^*(g)$ denotes the set of indices of control samples with gene g expressed in normal range according to inter-quartile range (IQR) definition [1]; outliers are defined to be the values greater than $q_3 + 1.5 \cdot (q_3 - q_1)$ or less than $q_1 - 1.5 \cdot (q_3 - q_1)$, where q_1 and q_3 denote the first and third quartiles. The final value of a gene g in sample i is defined based on the scaled value z_{gi}:

$$z^*_{gi} = \min\{|z_{gi}| + 1, z_{max}\},$$

where the upper limit z_{max} is used to prevent a single value becoming too dominant and is defined as the upper outlier limit in the IQR definition over the whole scaled data matrix. The constant 1 is added to $|z_{gi}|$ in order to avoid values less than 1. With this measurement processing, value z^*_{gi} is low if it is close to a typical control measurement whereas a higher value follows from a measurement different from a typical control sample.

1.2 Pathway preprocessing (Step 2)

Step 2 involves pathway preprocessing where some of the nodes and relations are dropped out from the pathways. More specifically, only nodes of type "gene" or "group" and relations between them are retained; others are filtered out from pathways since gene expression data provides no information about them. Further, if a pathway includes multiple nodes with identical Entrez id (or ids), those nodes are merged. For nodes of type "gene", one Entrez id is used to represent the node, which is selected based on the highest (unscaled) mean value among the control samples. Relations with identical start nodes and identical end nodes are merged and relations from a node to itself are removed.

Next, statistical and structural information is extracted from the pathways. For every node n in pathway P, we calculate the number of neighbour nodes denoted as $\#N_n^1$, the number of second neighbour nodes (only grandparent and grandchild nodes are counted) $\#N_n^2$, and the number of times a node with the same Entrez id(s) occurs in different pathways, denoted as $occ(n)$. For a node n in a pathway P, an importance factor is then calculated:

$$Imp(n, P) = \frac{sn(n, P)}{so(n, P)}, \qquad (1)$$

where $sn(n, P)$ denotes the weighted sum of direct (weighted by 1) and second (weighted by 0.5) neighbours of the node scaled to interval (0,1] within the pathway:

$$sn(n, P) = \frac{\#N_n^1 + 0.5 \cdot \#N_n^2}{\max\limits_{m \in P}\{\#N_m^1 + 0.5 \cdot \#N_m^2\}},$$

whereas $so(n, P)$ denotes the occurrences of the node scaled to interval (0,1] within the pathway:

$$so(n, P) = \frac{occ(n)}{\max\limits_{m \in P}\{occ(m)\}}.$$

In case the pathway is totally unconnected, i.e. it does not include any relations, all scaled neighbour values (sn) are set directly to 0 in order to avoid dividing by 0. Importance factor, defined as in equation (1), gets a high value if the node n has many neighbours (i.e. is well connected) in pathway P and does not appear in multiple pathways. Finally, in order to

keep the effect of importance factor moderate compared to the effect of the expression level, the importance factors are scaled using a logistic function so that median importance is 1; these scaled node importance factors are notated as $Imp^*(n, P)$.

1.3 Pathway node values (Step 3)

Step 3 calculates the pathway node values. First, each node n is given an initial value which is the average of all processed values z^*_{gi} mapped to the node. Note that for "gene" nodes, there is just one Entrez id mapped to the node, so "gene" nodes get directly that value. Nodes without any measured Entrez id initially get a value of -1. The initial value of node n for sample i is notated as u_{ni}.

After initializing the nodes, their values are modified based on a feedback phenomenon. If a node has a moderate measured value but all its child nodes have a large measured value, it suggests that the node has been active or inactive but later steps in the signaling may have inhibited/activated it back to a normal level. Therefore, the value of node n after feedback is defined as maximum of its initial value and weighted mean of the initial values of its child nodes. The weights of the child nodes are based on their occurrence so that child nodes appearing in multiple pathways have less weight. The value of node n in sample i after feedback is defined as

$$u^*_{ni} = \max \left\{ u_{ni}, \frac{\sum\limits_{m \in N_n^{child}} \frac{1}{occ(m)} u_{mi}}{\sum\limits_{m \in N_n^{child}} \frac{1}{occ(m)}} \right\},$$

where N_n^{child} is the set of child nodes of node n. Nodes with the initial value -1 are ignored.

1.4 Sample-specific pathway scores (Step 4)

Step 4 calculates pathway scores from the node values for each sample separately. For each node n in a pathway P the node values u^*_{ni} across all samples are ranked and these ranks are used as new node values u^r_{ni}. The advantage of ranks is their ability to emphasize delicate differences between samples. In case a node is not measured for some of the samples, the ranks of the measured samples are scaled to the full interval [1, number of samples]. A pathway score of pathway P for sample i is then defined as:

$$S(P,i) = \frac{\sum_{n \in P} u^r_{ni} \cdot Imp^*(n,P)}{\#P},$$

where $\#P$ is the number of nodes in pathway P. Dividing by $\#P$ is required as otherwise large pathways will have higher scores than small ones.
