# Supplementary material for: PASI: A novel pathway method to identify delicate group effects
Source: PLoS One. 2018 Jul 5;13(7):e0199991. doi: 10.1371/journal.pone.0199991 (PMC6033442; doi:10.1371/journal.pone.0199991)
Supplement: S1 Fig — (PDF) [file pone.0199991.s004.pdf]

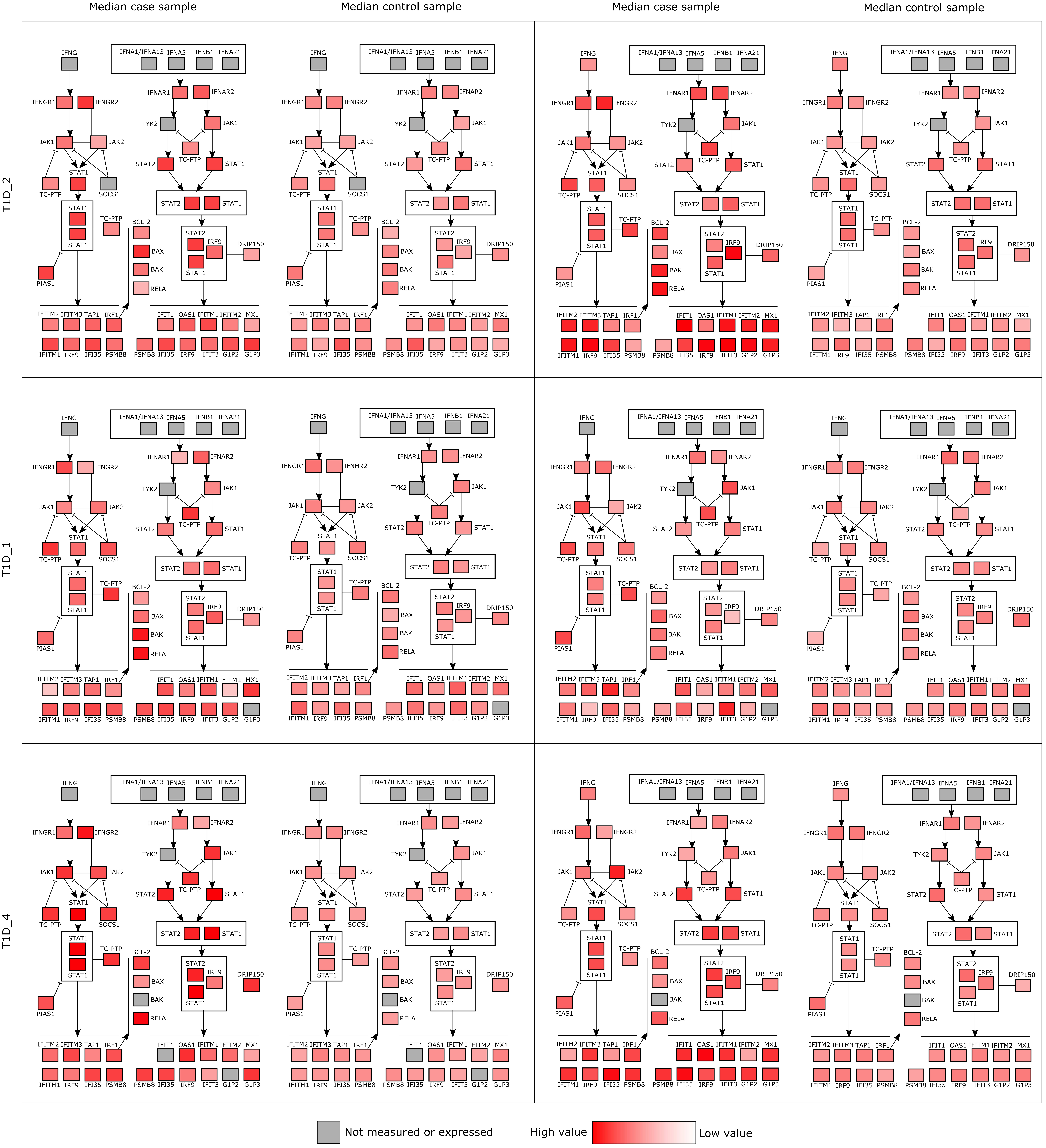

Interferon signaling pathway with genes colored according to case median sample (left hand side) and control median sample (right hand side) in tested data sets. The values used for coloring are scaled values with light color indicating control-like expression.
